# The need to pay attention to teamwork in pre-hospital emergency 

**Published:** 2019-08

**Authors:** Mostafa Soleimani, Abbas Dadashzade, Jafar Khani

**Affiliations:** ^*a*^Tabriz University of Medical Science, Tabriz, Iran.

## Abstract

**Background:**

The importance of teamwork to improve the quality of care and overcome the complexity of health system is clear to everyone. Therefore the current study aimed to review the studies in the field of team-work assessment in hospital and pre-hospital emergency.

**Methods:**

In this review study, scientific texts with the key-words of teamwork, paramedic, emergency medical technician, emergency department and inter-professional practice were searched in Google scholar, PubMed, scientific information database(SID), and Magiran.

**Results:**

Several studies have been found on the training and evaluation of teamwork in different parts, particularly in the treatment section. The results show that training and promotion of teamwork in health care lead to better performance and results, reducing clinical errors and reducing patient long length of stay In wards especially in the emergency department. Studies indicate that organizational support and teamwork training has the greatest impact in teamwork facilitate. In the pre-hospital emergency, there was a wide variety of teammates and less familiarity with teammates in EMS is associated with more occupational injuries.

**Conclusions:**

According to the aid team of pre-hospital emergency and their interaction with the hospital emergency department and other relief teams, training and evaluation of teamwork is important in EMS. However, there are few studies on teamwork in pre-hospital emergency especially in Iran. According to the importance of pre-hospital emergency mission, it is necessary to pay attention to teamwork in the emergency training curriculum and evaluation of teamwork in pre-hospital EMT.

**Keywords:**

Teamwork, Pre-hospital, Emergency, Medical technician

